# Breast cancer: from “maximum tolerable” to “minimum effective” treatment

**DOI:** 10.3389/fonc.2012.00125

**Published:** 2012-10-08

**Authors:** Umberto Veronesi, Vaia Stafyla, Alberto Luini, Paolo Veronesi

**Affiliations:** Department of Senology, European Institute of OncologyMilan, Italy

**Keywords:** breast cancer, randomized trials, maximum tolerable treatment, minimum effective treatment, conservative treatment

## Abstract

Randomized trials have played a fundamental role in identifying better treatments for most type of diseases, especially in the oncological field. In breast cancer, the shift from “maximum tolerable” to “minimum effective” treatment has been evident since the 1970s and has been based on the results of international randomized trials. The progress of breast surgery represents an excellent model of the evolution of science and the aim of this article is to review the main randomized studies that changed everyday practice in breast surgery.

Medical science has gone through an important evolution in the past century that was based on the progress in research and on the advances in technology. The field of surgical oncology is important, as surgery represents the most common and essential treatment for solid tumors. In the last 50 years a lot of effort was made to improve the treatment modalities, in order not only to prolong the survival, but also to ensure a good quality of life. The advances achieved are greatly attributed to the clinical research. The aim of clinical research is the collection of evidence in order to establish the best treatment and this is achieved through clinical trials. They may be observational studies, when the results of a treatment procedure are compared with the historical controls. Randomized trials are the most valid and the most commonly used in surgical oncology, as they give information regarding the efficacy of a new procedure by comparing homogeneous groups of patients who undergo different treatments.

The first randomized trial was published in 1948 in British Medical Journal and reported on the effects of streptomycin on pulmonary tuberculosis (1948). Throughout the years this type of clinical trial was established as the gold standard of evidence based medicine. In 1996, the guidelines for reporting randomized trials were published for the first time and were updated in 2010 (Altman, 1996; Schulz et al., 2010). Simple Randomization, similar to “fair coin-tossing,” was the most commonly used in the early periods mainly for trials with up to 200 participants (Schulz and Grimes, 2002). Today, for larger trials, the restricted randomization is preferred, in order to guarantee group sizes balance. Another important parameter is the allocation concealment. According to this procedure, the participant and the researcher are not aware of the treatment allocation, until after the patient enters the study, thus ensuring complete impartiality. Possible disadvantages of a randomized trial are high cost, long duration, and limited external validity, as the results cannot always be widely applied outside the participating centers. From the ethical point of view, an informed consent signed by the participant is mandatory, as well as the approval of the study by the hospital ethics committee. Every participant has to be thoroughly informed about the possible treatments, he will or will not receive, and about their possible side effects and implications. The task of ethics committee or the institutional review board (IRB) is to approve or disapprove, and to monitor the course of the trial bearing into consideration the rights of the participants. When all the necessary criteria are met, the analysis of the data provided by a randomized trial gives evidence on Therapy/Prevention and Harm according to the Oxford Centre for Evidence-based Medicine (2010).

## From Halsted mastectomy to breast conservation

Randomized trials have played a fundamental role in identifying better treatments for most type of diseases, especially in the oncological field. Although, it is statistically easy to compare two or more treatments, with an expected quantitative difference at the endpoint -usually the survival-, it is much more difficult to compare two treatments that are not expected to be quantitatively different. This is relevant for studies that aim at evaluating improvement in quality of life offered by conservative treatments. In this case no difference in survival is expected, but qualitative parameters, such as quality of life, which are however, difficult to be quantified. These trials, named “equivalence trials,” have an important role to proceed toward less aggressive treatments. This new objective became important after a change in paradigms in cancer treatment was proposed to the medical community in the 1970s. The change referred to the substitution of the traditional paradigm of “maximum tolerable treatment” to the new opposite paradigm of “minimum effective treatment.” Since the 1970s the paradigm has been applied to many instances and the evolution of treatment of breast cancer is an excellent model. The review of this model is the aim of this paper.

Randomized trials have contributed significantly to practice changing in surgical oncology in the last 50 years. In the field of breast surgery, the conduction of randomized trials was proven to be vital for all the revolutionary changes performed. The passage from “maximally tolerated” to “minimally effective” treatment has not been easy and the idea of conserving a large portion of an already affected organ was challenged by many surgical oncologists. It was only the large randomized trials performed in breast cancer patients that made possible the acceptance of breast conservation, and led to a complete modification of the principles of breast surgery. Over the years, Halsted mastectomy was replaced by lumpectomy or quadrantectomy, and external high energy radiotherapy was an integrated component of treatment. An attempt to improve the prognosis through more extended treatments was the aim of the trial on internal mammary node dissection. A large randomized trial was published in 1976 and it was an international trial comparing radical mastectomy with and without internal mammary dissection (Lacour et al., 1976). From 1963 to 1968, 1453 patients in 5 breast centers were randomized. The 5 years survival was similar in the two groups and only patients with medially located tumors and positive axillary nodes were shown to moderately benefit from internal mammary dissection. The Cancer Institute of Milan participated in this study and published the 10-years follow up of 716 patients in 1981 (Veronesi and Valagussa, 1981). Overall survival (OS) and disease free survival were equal in the two groups. There was no difference in recurrence rates on the operating field, the axilla and the supraclavicular fossa. The radical mastectomy group had higher parasternal recurrences compared to the group with the extended operation, which; however, were relatively low (3.7%). The 10-years update of the multicenter study that was published 2 years later confirmed no difference in survival and in relapse-free survival (Lacour et al., 1983). This first trial attempted to explore the impact of more aggressive surgery than the standard and failed the goal.

Thereafter, the trend went to the opposite direction, to compare radical mastectomy with less mutilating interventions, and to investigate the role of radiotherapy in the local control. The Milan I trial (1973–1980) randomized 701 pT1 breast cancer patients into either Halsted mastectomy or quadrantectomy with axillary dissection and breast radiotherapy. The first results were published in 1981 and showed no difference in disease-free (DFS) and OS (Veronesi et al., 1981). The study update with a 20-years follow up confirmed the preliminary findings, establishing the concept of breast conservation as a standard of care (Veronesi et al., 2002). In the same period in France, 179 patients were randomized to either modified radical mastectomy or tumorectomy with axillary sampling and breast irradiation (Sarrazin et al., 1989). Both groups were shown to have similar OS, DFS, and LRR at 10 and 15 years (Arriagada et al., 1996).

In 1974, another randomized trial begun in the United States, the NSABP B-04, that recruited 1079 breast cancer patients. In case of clinically negative axilla, they were randomized to either total mastectomy, with axillary radiotherapy or to total mastectomy with delayed axillary dissection, in case of appearance of clinically positive axillary nodes. Patients with clinically positive nodes were randomized to either radical mastectomy or total mastectomy with axillary radiotherapy. No benefit in OS and DFS survival was found from radical mastectomy on the 10-years update (Fisher et al., 1985b).

In 1976, the NSABP B-06 trial started randomizing patients to total mastectomy, lumpectomy alone or lumpectomy with breast radiotherapy (Fisher et al., 1985a, 1989, 2002). Based on the accrual of 1851 patients, OS and distant DFS were similar between the three groups, but radiotherapy was shown to reduce the breast recurrence rate after lumpectomy.

Four years later, in 1979, the National Cancer Institute conducted a prospective randomized study comparing modified radical mastectomy vs. lumpectomy—with positive or negative resection margins- with axillary dissection and adjuvant radiotherapy (Lichter et al., 1992). After 20 years of follow up of 237 patients, OS and DFS were comparable, however, according to the authors “long-term in-breast failures continued to occur throughout the follow up” (Poggi et al., 2003).

A study with a similar design was launched in 1980 by the EORTC. The 10801 trial randomized 868 patients with T1 and T2 tumors until 1986 to either modified mastectomy or lumpectomy –with positive or negative resection margins- with axillary dissection and adjuvant radiotherapy (van Dongen et al., 2000). At 10 years, the results were similar to those of the NCI trial. OS and distant metastasis-free survival were similar; however, local recurrences were statistically higher in the lumpectomy group.

Between 1983 and 1989 the Danish Breast Cancer Cooperative Group after randomizing 905 patients to either modified radical mastectomy or lumpectomy with axillary dissection and radiotherapy, concluded that OS and DFS did not differ significantly (Blichert-Toft et al., 1992).

These large randomized trials conducted in the 1970s and early 1980s showed the way to “less surgery” and practically changed the route of breast cancer surgery. Furthermore, they confirmed the hypothesis that the prognosis of breast cancer patients is linked to the presence or absence of distant metastasis and changes in local treatment do not affect the OS. Breast conservation became not only a viable option, but a standard treatment, and those trials updates published in the beginning of the twenty-first century confirmed that mutilating interventions such as radical mastectomy belong to the past. However, some uncertainty remained about the extent of the breast conservation. This issue was further investigated with a randomized study (Milan II) that was conducted between 1985 and 1987 and its results were published in 1990 (Veronesi et al., 1990). Seven hundred and five patients with tumors up to 2.5 cm were randomized to receive either quadrantectomy or lumpectomy. All patients underwent axillary dissection and radiotherapy. In quadrantectomy, 2–3 cm of normal tissue surrounding the tumor was excised, as well as the tumor overlying skin and the underlying fascia. In lumpectomy, only a rim of 1 cm around the tumor was excised. After a follow up of 10 years OS and distant metastasis rate were not different, while in breast tumor recurrence was significantly higher in the lumpectomy group (Mariani et al., 1998).

Following the establishment of breast conservation as treatment of choice for early breast cancer, the role of radiotherapy on loco-regional control remained to be clarified. Light was shed on the effect of adjuvant radiotherapy by two randomized trials. The first was conducted at the Milan Cancer Institute (Milan III) between 1987 and 1989 and recruited 567 patients with tumors up to 2.5 cm (Veronesi et al., 1993). They were randomized to quadrantectomy with axillary dissection with or without adjuvant radiotherapy. The radiotherapy group had a significantly lower local recurrence rate; however, the 5-years OS was comparable. Similarly, the Uppsala-Orbero Breast Cancer Study Group, reported the same conclusions in a study of 381 patients with pT1 tumors (Uppsala-Orebro Breast Cancer Study Group, 1990). Radiotherapy is nowadays considered a component of breast conservation, at least in women who are younger than 60 years old. For patients over 60 years old, a multicenter prospective randomized trial was conducted, in order to assess the necessity of radiotherapy. Between 2001 and 2005, 749 patients with early breast cancer were assigned to either surgery only or to surgery and breast radiotherapy and after 5 years of follow up there was found a difference in in breast recurrence (2.5% vs. 0.7%), but no difference in OS and in distant disease free survival (Tinterri et al., 2009).

## Conservation of axillary nodes

The concept of “less surgery” was extended to the treatment of the axilla. The role of radiotherapy on the axilla was evaluated in a study conducted in Milan between 1995 and 1998 (Veronesi et al., 2005). Four hundred and thirty five patients with small tumors were randomized to either axillary radiotherapy or nothing. After 63 months of follow up, the axillary metastases presented were lower than expected in both groups, suggesting that axillary dissection can be avoided in this subgroup of patients and that radiotherapy has a protective effect.

The introduction of the sentinel lymph node biopsy put further under investigation the role of axillary dissection. It was already anticipated that the positivity of the axilla was an element of prognosis and not a reason to perform more extensive surgery. Sentinel lymph node biopsy is a method of “predicting” the axillary status sparing the patient from axillary dissection and its often devastating complications, like arm lymphedema. As soon as the technique of sentinel lymph node biopsy was standardized, a series of randomized control studies started worldwide. The first was the Milan Trial, that in 1998 and 1999 randomized 506 patients with tumors up to 2 cm to two arms, one receiving immediate axillary dissection and the other receiving the dissection only if the sentinel node was involved (Veronesi et al., 2006). After 79 months of follow up, OS and DFS were equal. Only one case of axillary recurrence was observed among the patients in the SLN group who did not receive axillary dissection, although eight false negatives would be expected. The long term analysis showed that patients had less mortality rates after sentinel lymph node biopsy policy than after immediate dissection (25 vs. 18 deaths).

An identical study was conducted between 1999 and 2004, that randomized 5611 women with invasive breast cancer up to 4 cm from 80 centers in the USA and in Canada to either axillary dissection or to sentinel lymph node biopsy alone with axillary dissection only if the SLN was positive (Krag et al., 2010). After 95.6 months of follow up, OS and DFS were similar in the two groups. A sub-study reported that up to 12 months postoperatively, patients with axillary dissection had significantly higher arm morbidity and significantly more restricted work and social activity and impaired QoL (Land et al., 2010).

The ALMANAC trial, is a multicenter UK trial, that studied the QoL in patients with SLN vs. axillary dissection between 1999 and 2003 (Mansel et al., 2006). One thousand and thirty one patients participated and at 12 months it was evident that lymphedema and sensory loss were higher in the axillary dissection group; operative time, drainage use, hospitalization, and resumption of normal life was much longer in axillary dissection group, while in SLN group, patients had a higher QoL and arm functioning scores. The results of the SNAC trial and the Danish Breast Cancer Cooperative Group confirmed the ones of ALMANAC (Gill, 2009). Arm lymphedema and dysfunction were significantly higher in the axillary dissection group at 12 months for ALMANAC and at 18 months for DBCCG (Husted Madsen et al., 2008).

The outcomes of these studies made clear that in case of non-metastatic disease to the axilla, axillary dissection is not only unnecessary, but also harmful. But what if the axillary lymph nodes are positive? Is axillary dissection still necessary or can it be avoided? The answer to this question, that takes conservative treatment of breast cancer one step forward, is nowadays under investigation. The NSABP Z0011 trial has randomized 891 patients with T1 and T2 tumors and positive SLN from 115 centers from 1999 to 2004 to receive axillary dissection or no further treatment (Giuliano et al., 2011). At 6.3 years of follow up, the 5 years OS and the DFS were not different in the two groups, suggesting that in this subgroup of patients, axillary dissection may not be necessary. The EORTC 10981 AMAROS trial has randomized patients with positive SLN to either axillary dissection or axillary radiotherapy from 2001 until 2010 and its results are still to be published (Rutgers et al., 2004). Another multicentric randomized trial studying the role of axillary treatment is the SOUND trial that started at the beginning of this year at the IEO, in Milan. Patients with pT1 tumors and negative axillary US scan are randomized to both SLN biopsy and axillary dissection if positive or to no sentinel biopsy at all. The results of this trial are awaited, as it might completely change the approach to the axillary treatment, abandoning the sentinel node biopsy in patients with an uninvolved axilla at clinical and ultrasonographical examination.

## Breast radiotherapy

Adjuvant radiotherapy was shown to be an important element to breast conservation. Breast radiotherapy has followed the same course as breast surgery from large fields and high doses to fields and doses as limited as possible. Recently, the concept of accelerated partial breast irradiation has emerged and positive experience is being accumulated. An interesting modality was to anticipate the radiotherapy, which could be delivered during surgery, intraoperative radiotherapy (IORT). Two large randomized IORT trials are ongoing at the moment, the ELIOT trial and the TARGIT trial. In ELIOT protocol electrons are delivered in the quadrantectomy site, while in the TARGIT protocol low energy X rays are used in the lumpectomy site after the tumor resection and while the patient is still under general anesthesia. The first results of ELIOT protocol show that pulmonary fibrosis is significantly less in IORT patients compared with external radiotherapy patients (Rampinelli et al., 2011). The TARGIT trial results show that of 1113 patients who were randomized to either IORT or external beam radiation, at 4 years of follow up 6/996 in the IORT group and 5/1025 in the external radiotherapy group developed a local recurrence (Vaidya et al., 2010). Complications and major toxicity was similar in the two groups, while radiation toxicity was significantly lower for IORT. The TARGIT trial outcomes are encouraging and, if long term follow up, along with ELIOT trial results, confirms the non-inferiority of IORT, it will be another step in the evolution of breast cancer treatment.

## Conservative mastectomy

Conservative mastectomy is the last step in breast cancer conservative treatment. It combines total excision of breast parenchyma sparing the skin and the nipple areola complex, thus offering a very good aesthetic result by preserving the patient's body image thanks to the immediate reconstruction of the breast. Randomized controlled trials are not available on this emerging technique, and so far the only available data in the literature comes from cohort series with heterogeneity on indications and reconstructive techniques. The outcomes on oncologic safety are satisfactory, as is the cosmesis, according to surgeons' and patients' evaluation. In our Institute, the European Institute of Oncology, we have the largest series of patients treated with conservative mastectomy with OS rates similar to those of skin sparing mastectomy and with superior aesthetic outcomes (Petit et al., 2009). At the moment we are considering a randomized trial on patients treated with conservative mastectomy focusing on IORT on the conserved nipple areola complex, in order to evaluate the sterilizing effect of radiotherapy on nipple recurrence.

## Conclusions

The large number of randomized trials conducted in the last 50 years has completely overturned the radio-surgical breast cancer management. The long term beneficial effects were evident especially with regards to the motivation of women to early detection. Women know that the early discovery of a “small nodule” on the breast will not only save their life, but will make it possible to preserve their body image. In the western world, most women participate in many large randomized programs for early detection of breast cancer and the evolution of images (mammography, ultrasound, and MRI) has greatly facilitated this progress.

### Conflict of interest statement

The authors declare that the research was conducted in the absence of any commercial or financial relationships that could be construed as a potential conflict of interest.
